# The Unnecessary in Medicine and Surgery

**Published:** 1882-04

**Authors:** S. V. Clevenger

**Affiliations:** Chicago


ARTICLE II.

### THE UNNECESSARY IN MEDICINE AND SURGERY.

BY S. V. CLEVENGER, M.D., CHICAGO.

Therapeutists, who deserve the name of teachers, wisely refuse to load down their pupils with formularies of their own or other's making. Many of the youngsters consider it a hardship that they should be compelled to construct their own prescriptions ; but if they are capable of thinking at all, it will not be long before they will have occasion to thank the old professor for thus putting them at work with their brains, eliminating incompatibles, compounding eligibles, and omitting the unnecessary from their recipes. Were this course pursued by all instructors, such things as polypharmacy and irrationalism would be confined to the practice of the uneducated. Giving medicine without a definite idea of what the giver expects to accomplish by its administration is no worse than the practice of ordering ingredients the uses of which are unknown to the prescriber, but which have the sanction of "authority." He who resorts to formularies, habitually, belongs to a class the least informed of which could see nothing grotesque in the witch's broth of newt and frog members, or the materia medica in Bacon's *Instauratio Magna,* such as the scrapings from murderers' skulls, etc.

The student who precedes his ordinary medical course with a year's training in practical inorganic and organic chemistry and pharmacy brings to his aid, in his therapeutic studies, an intellect from which the cobwebs and rubbish of past ages have been swept away. He is not apt to commit serious blunders in his private practice, much less publish an outrageous formula, as I have known truly and worthily eminent physicians to do, owing to the fact that a knowledge of the branches mentioned was not considered indispensable at their schools and in their early days. I have many chemists and pharmacists among my friends in this city, and were I to base an opinion of the general abilities of prescribers upon the prescriptions shown to me by the druggists it would be in the main unjust. Pages of this journal could be filled with ludicrous formulæ from such sources, the most provoking feature being the fact that many of these prescriptions are faithful copies of printed originals—sometimes even the typographical errors have been transcribed with Chinese fidelity.

The method of giving single remedies, in their proper menstrua, if any needed, or with necessary vehicles and corrigents and avoiding the old-fashioned *olla podrida* mixtures, would indicate a desire, at least, on the part of the physician adopting it, to be rational in his treatment.

The abolition of the unnecessary in medicine as in surgery can only be brought about by calling thought to the aid of the healing art. The consequences of want of reflection are often more apparent in the surgeon's work than in the physician's, as one little instance may go to show. I recently assisted an undeniably skilful gynæcological surgeon in the closure of a vesico-vaginal fistula. The doctor has been in the habit of using tenacula in such cases to hold the labia apart. With trained assistants there might be certain advantages in so doing, but as one of the aids in this case was an undergraduate, without previous experience, though very intelligent, he unfortunately caused an unnecessary amount of laceration on the side of the vagina he undertook to hold away with his tenaculum during the operation, and finally succeeded by an undue amount of traction in tearing an artery the inaccessibility of which occasioned almost as much trouble in stopping the hemorrhage as did the vesico-vaginal operation. At first I was inclined to blame the student for his want of care, but now think that the use of tenacula at all was unnecessary when a Sims' speculum, spatula, or even the finger of an assistant might have answered as well.

Similarly the use of hooks in patellar fractures or the tenaculum in drawing the tongue forward when respiration is interfered with in anæsthesia, it seems to me, could be dispensed with. In the latter case the tongue can be firmly grasped and held as well by a dry handkerchief between the fingers.

At a clinic a few years ago a professor of gynæcology, with his patient before him on the operating chair, began his lecture to the students assembled about him, thus : "Gentlemen, the neck of the uterus is very poorly supplied with sensory nerves, and I have the means at hand of demonstrating this fact to you. In bringing the os into view through the speculum, I shall use this tenaculum, which she will not feel at all." Here a comparatively large hook was stabbed into the cervix, instantly followed by the woman vociferating Ouch !

While it might not be advisable to give one's tenacula to the nearest butcher, certainly less harm is likely to be done were instruments used which would cause no laceration or pain when such instruments answer equally well.

